# Efficacy and safety of air-flow beds for autologous hematopoietic stem cell transplantation in multiple myeloma: a retrospective cohort study

**DOI:** 10.3389/fonc.2026.1953570

**Published:** 2026-09-30

**Authors:** Zhangbiao Long, Dinghui Zhao, Min Ruan, Yutong Jin, Yuxin Li, Zhengfeng Hou, Jian Hong, Jifei Dai, Xinglin Liang, Qingsheng Li, Jian Ge

**Affiliations:** 1 Department of Hematology, The First Affiliated Hospital of Anhui Medical University, Hefei, China; 2 School of Pharmacy, Anhui University of Chinese Medicine, Hefei, China

**Keywords:** air-flow bed, autologous stem cell transplantation, cost savings, infection control, multiple myeloma

## Abstract

**Background:**

Autologous hematopoietic stem cell transplantation (ASCT) is the standard consolidation therapy for multiple myeloma (MM), but its widespread implementation is constrained by the high cost and limited availability of conventional laminar airflow rooms. The air-flow bed, a single-bed unit equipped with high-efficiency particulate air (HEPA) filtration providing class 100 (ISO class 5) air quality, represents a more accessible and cost-effective alternative.

**Methods:**

In this retrospective cohort study, we compared the efficacy, safety, and costs of ASCT performed in air-flow beds (Group A, n=91) versus conventional laminar airflow rooms (Group B, n=60) in 151 MM patients treated between 2022 and 2026. Multivariable regression models were adjusted for potential confounders. Sepsis was analyzed descriptively because of the small number of events. Platelet transfusion requirements were analyzed using negative binomial regression.

**Results:**

Engraftment outcomes were comparable between groups. Group A showed significantly lower rates of mucositis, diarrhea, and nausea/vomiting, and no sepsis events occurred in Group A compared with four cases (6.7%) in Group B. Pathogen detection rate was lower in Group A than in Group B (5.5% vs. 15.0%, P = 0.050). In negative binomial regression, air-flow bed use was independently associated with a lower number of platelet transfusion units (IRR = 0.50, P<0.001). Higher CD34+ cell dose was also independently associated with fewer platelet transfusions (IRR = 0.94 per 1×10^6^/kg increase, P = 0.003). Sepsis occurred exclusively in Group B (4/60, 6.7% vs. 0/91, 0%). With only four events and complete separation between groups, multivariable logistic regression was not statistically reliable. This finding is therefore reported descriptively and no adjusted effect estimate is presented. Median total costs were significantly lower in Group A (¥121,738 [approximately $16,908] vs. ¥137,506 [approximately $19,098], P = 0.0002). No transplant-related mortality occurred in Group A, whereas one patient (1.7%) in Group B died.

**Conclusion:**

These findings suggest that air-flow beds may represent a safe, effective, and economically viable alternative to conventional laminar airflow rooms for ASCT in MM. Because protective environment and transplant era were strongly intertwined, these observational associations should be interpreted with caution and regarded as hypothesis-generating. Prospective validation in randomized controlled trials is warranted to confirm these observations.

## 1 Introduction

Autologous hematopoietic stem cell transplantation (ASCT) has long been a cornerstone in the treatment of eligible patients with newly diagnosed multiple myeloma (MM), significantly prolonging progression-free survival (1–3). The procedure involves mobilization and collection of peripheral blood stem cells following chemotherapy-based or steady-state mobilization, followed by high-dose conditioning chemotherapy such as melphalan, reinfusion of the cryopreserved stem cell product, and subsequent hematopoietic recovery (4–6).

The widespread implementation of ASCT for MM remains constrained by infrastructure requirements. Conventional laminar airflow rooms, which provide a sterile environment during the neutropenic period, entail substantial capital investment and are therefore available only in major tertiary centers (7). However, the effectiveness of high-efficiency particulate air (HEPA) filtration in reducing transplant-related mortality remains controversial (8, 9). Moreover, these isolation rooms impose a closed environment that restricts family visitation and personal caregiving, contributing to considerable patient psychological distress. These limitations underscore the urgent need for alternative protective strategies that are both safe and more accessible.

Outpatient ASCT has been explored as a potential solution to circumvent these infrastructure barriers. Recent studies have demonstrated that outpatient mobilization chemotherapy for MM is safe and feasible, with substantial cost savings and improved bed utilization without compromising patient safety (10–12). A machine learning-based analysis of 109 MM patients suggested that risk-stratified outpatient protocols could reduce bed usage without compromising safety, though accurate prediction of neutropenic fever remains challenging (13). However, this approach raises major safety concerns, as immunocompromised patients undergoing ASCT are at substantially increased risk for severe life-threatening infections when managed outside controlled protective environments.

The air-flow bed represents a promising alternative to conventional laminar airflow rooms. This single-bed unit is equipped with a HEPA filtration system that provides class 100 (ISO class 5) air quality equivalent to that of full-room laminar flow facilities, yet it can be installed in standard hospital wards at a fraction of the cost. Unlike traditional isolation rooms, the air-flow bed allows family members to accompany patients throughout hospitalization, offering meaningful psychosocial support during the prolonged period of bone marrow suppression (14). Emerging evidence from alternative protective environment models, including early step-down from HEPA-filtered rooms to non-HEPA wards and plasma sterilizer environments, suggests that comparable infection control and engraftment outcomes may be achievable without full-room laminar flow facilities (15, 16). Together, these findings indicate that HEPA filtration alone may suffice for infection control, and patient-centered factors such as family presence may offer additional benefits without compromising safety.

Despite these promising observations, data directly comparing air-flow beds with conventional laminar airflow rooms in the specific context of ASCT for MM remain scarce. In this retrospective cohort study, we analyzed 151 patients with multiple myeloma who underwent ASCT at our center between February 2022 and March 2026, of whom approximately two-thirds were managed in air-flow beds. We aimed to evaluate the feasibility and safety of air-flow beds for ASCT in MM by comparing engraftment outcomes, adverse events, and hospitalization costs against those of conventional laminar airflow rooms.

## 2 Methods

### 2.1 Study design and patients

This was a retrospective cohort study conducted at the Department of Hematology, The First Affiliated Hospital of Anhui Medical University. Consecutive patients with multiple myeloma who underwent autologous peripheral blood stem cell transplantation (ASCT) between February 2022 and March 2026 were identified from an institutional database. Patients were classified into one of two groups according to the protective environment used during transplantation: Group A (air-flow bed, n=91) or Group B (conventional laminar airflow room, n=60). The air-flow bed is a single-bed unit equipped with a high-efficiency particulate air (HEPA) filtration system that provides class 100 (ISO class 5) air quality, and is placed in a standard ward setting (**Figure 1**; **Supplementary Figure 1**). The conventional laminar airflow room is a dedicated isolation room with full-room HEPA filtration and positive pressure ventilation.

**Figure 1:**
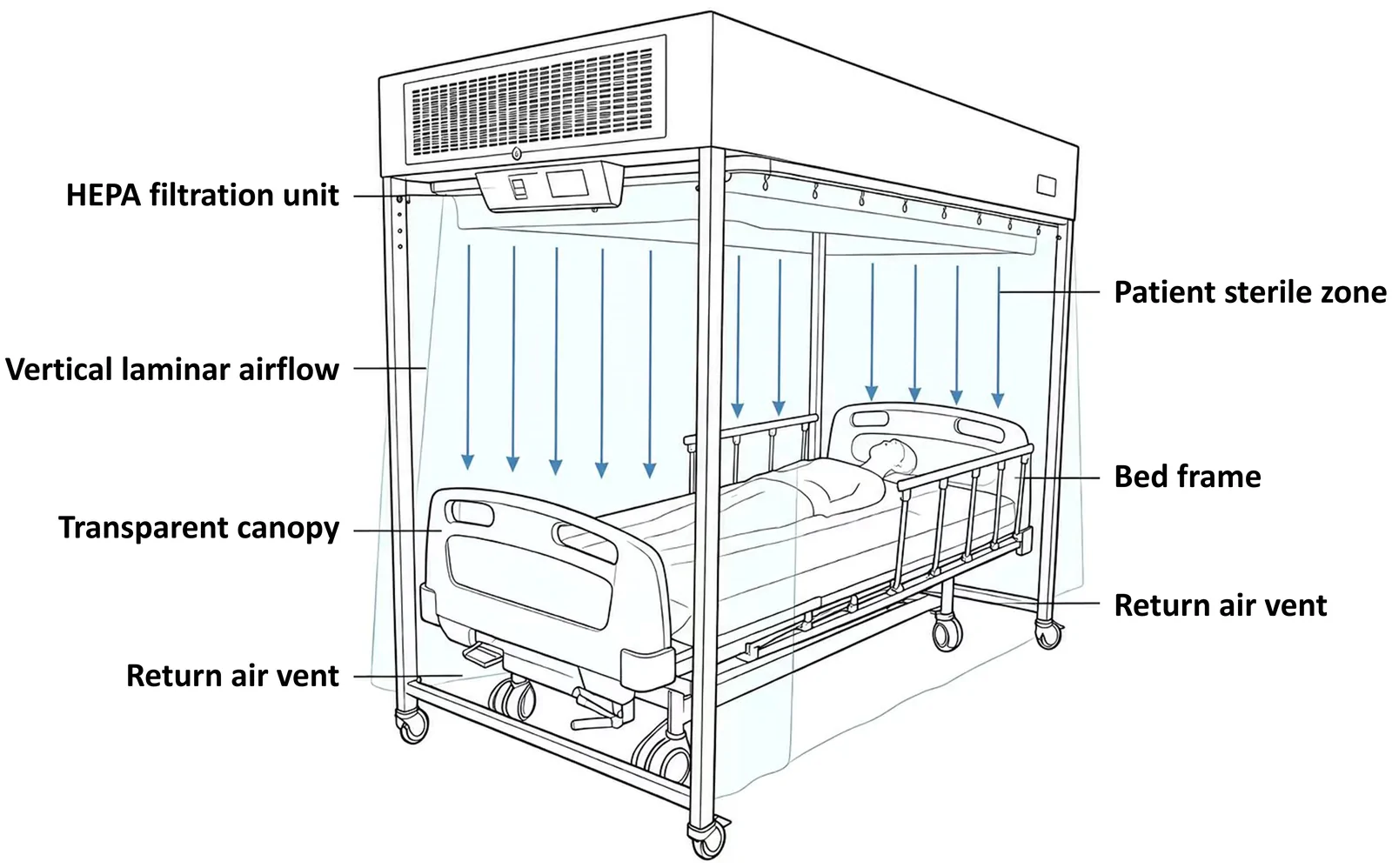
Schematic diagram of the air-flow bed system. The figure illustrates the structure and working principle of the air-flow bed, including the HEPA filtration unit at the top, the vertical laminar airflow pattern indicated by downward arrows, the transparent canopy surrounding the patient area, the patient positioned on the bed, and the return air vent at the bottom. The system delivers class 100 (ISO class 5) air quality over the patient area through unidirectional airflow.

Patient allocation was determined by evolving institutional practice rather than by patient characteristics or physician preference. From 2022 to 2024, air-flow beds were the predominant protective environment for autologous transplantation at our center, although a small number of patients (n=7) were managed in the existing conventional laminar airflow rooms during this period. Following the completion of new transplant wards in 2025, all subsequent autologous transplantations were performed in conventional laminar airflow rooms. The distribution of patients by calendar year is shown in **Table 1**. The same medical team managed all patients throughout the study period, with unchanged supportive care protocols.

**Table 1:** Baseline characteristics of patients undergoing autologous stem cell transplantation.

Characteristics	Whole cohort	Group A	Group B	P value
Age (year)	56.54 ± 6.51	55.95 ± 5.67	57.45 ± 7.58	0.1655
Gender				0.2404
Male	89	50	39	
Female	62	41	21	
Myeloma type				0.5579
IgG	80	45	35	
IgA	33	22	11	
IgD	2	2	0	
κ	17	12	5	
λ	16	9	7	
Non-secretory	2	1	1	
Biclonal	1	0	1	
ISS				0.5037
I	22	12	10	
II	65	37	28	
III	64	42	22	
R-ISS				0.9130
I	33	20	13	
II	78	48	30	
III	40	23	17	
Response status				0.9535
CR	79	47	32	
VGPR	50	31	19	
PR	21	13	9	
Mobilization method				0.0016
Steady-state	126	69	57	
Chemotherapy-based	25	22	3	
CD34+ cells (×10^6^/kg)	5.66 ± 3.51	6.15 ± 4.06	4.90 ± 2.26	0.0318
Conditioning Regimen				0.0048
Melphalan alone	128	71	57	
Melphalan+Bendamustine	23	20	3	
Melphalan dose (among Melphalan alone)				0.7940
Mel200	98	55	43	
Mel140	30	16	14	
Calendar year distribution				
02022	13	13	0	
2023	37	34	3	
2024	48	44	4	
2025	52	0	52	
2026	1	0	1	

Data are presented as mean ± SD, n (%), or median (IQR) as appropriate. Group A: air-flow bed (n=91); Group B: conventional laminar airflow room (n=60). P values were calculated using the chi-square test or Fisher’s exact test for categorical variables, and the Student’s t-test or Mann-Whitney U test for continuous variables, as appropriate. CR, complete response; VGPR, very good partial response; PR, partial response; ISS, International Staging System; R-ISS, Revised International Staging System; SD, standard deviation; IQR, interquartile range.

Inclusion criteria were: (1) age 18 years or older; (2) diagnosis of multiple myeloma according to the International Myeloma Working Group (IMWG) criteria; (3) receipt of high-dose melphalan-based conditioning followed by autologous PBSC infusion; and (4) availability of complete medical records. Exclusion criteria were: (1) prior autologous or allogeneic stem cell transplantation; (2) concomitant malignancy; (3) active uncontrolled infection at the time of transplantation; or (4) severe cardiac, hepatic, or renal dysfunction precluding transplantation.

The study was approved by the Ethics Committee of The First Affiliated Hospital of Anhui Medical University. Written informed consent was obtained from all patients before stem cell collection, covering both the transplantation procedure and the use of clinical data for research purposes.

### 2.2 Stem cell mobilization and collection

Patients underwent either steady-state mobilization or chemotherapy-based mobilization according to physician discretion and institutional practice. For steady-state mobilization, recombinant human granulocyte colony-stimulating factor (G-CSF) was administered at a dose of 10 μg/kg per day for 4 to 5 days, with plerixafor (0.24 mg/kg subcutaneously) added on the evening of day 4 if the peripheral blood CD34+ cell count was below 20/μL. For chemotherapy-based mobilization, etoposide was administered at a dose of 375 mg/m² intravenously, followed by G-CSF at 10 μg/kg per day from day 5 until the completion of apheresis. Peripheral blood stem cells were collected using either the COM.TEC device (Fresenius-Kabi, Germany) or the Spectra Optia device (Terumo BCT, USA) in the autoMNC or continuous mononuclear cell collection mode (17, 18). The target CD34+ cell dose was 2.0×10^6^/kg or higher. The collected product was assessed for CD34+ cell count, viability, and sterility according to institutional standard operating procedures.

### 2.3 Transplantation procedure

All patients received high-dose conditioning therapy prior to stem cell infusion. The conditioning regimen consisted of melphalan at a dose of 200 mg/m², or 140 mg/m² for patients aged 65 years or older or those with renal impairment (defined as creatinine clearance below 60 mL/min), following institutional protocol based on EBMT guidelines, administered intravenously on day -2. The distribution of Mel200 versus Mel140 in each group is shown in **Table 1**. A subset of patients received a combination of melphalan and bendamustine (melphalan 140 mg/m² plus bendamustine 120 mg/m² on days -2 and -1) as part of an ongoing institutional pilot study exploring the feasibility of this combination in patients with high-risk cytogenetics or suboptimal response to induction. The conditioning regimen was selected at the discretion of the treating physician based on patient age, performance status, organ function, and disease characteristics (**Table 1**).

Following conditioning, the cryopreserved stem cell product was thawed rapidly in a 37 °C water bath and infused through a central venous catheter on day 0. All patients received standard supportive care, including antiemetics, proton pump inhibitors, and antimicrobial prophylaxis (fluoroquinolones, fluconazole, and acyclovir) according to institutional guidelines. G-CSF (10 μg/kg per day) was administered subcutaneously from day +5 until neutrophil engraftment. Transfusion support was provided for hemoglobin below 60 g/L or platelet count below 10×10^9^/L, or below 20×10^9^/L in the presence of fever or bleeding.

### 2.4 Protective environment

Patients in Group A were managed in air-flow beds placed in standard single-occupancy hospital rooms. The air-flow bed system (classified as class 100/ISO class 5) delivers HEPA-filtered vertical laminar air over the patient area. Patients were encouraged to remain within the bed area during the neutropenic period, but were permitted limited ambulation within the room. Family members were allowed to accompany patients throughout hospitalization, with appropriate isolation precautions including hand hygiene, mask-wearing, and restricted movement within the room. Healthcare workers entering the room followed standard contact and droplet precautions.

Patients in Group B were managed in conventional laminar airflow rooms equipped with full-room HEPA filtration and positive pressure ventilation (class 100/ISO class 5). Standard reverse isolation precautions were applied. In this setting, family visitation and bedside accompaniment were not permitted during the entire transplantation course. Communication between patients and family members was maintained through video calls. Healthcare workers entering the room followed full protective attire including gown, gloves, mask, and cap. Patient mobility was restricted to the room.

These differences in visitation policies, patient mobility, and healthcare worker exposure represent important contextual factors that may influence infection-related outcomes and are acknowledged as potential confounders in the Discussion.

### 2.5 Definitions and outcome measures

Engraftment: Neutrophil engraftment was defined as the first of 3 consecutive days with an absolute neutrophil count (ANC) of 0.5×10^9^/L or higher. Platelet engraftment was defined as the first of 7 consecutive days with a platelet count of 20×10^9^/L or higher without transfusion support (19). Primary engraftment failure was defined as failure to achieve ANC of 0.5×10^9^/L or higher by day 28 or platelet count of 20×10^9^/L or higher by day 42 (20).

Adverse events: Non-hematologic adverse events were graded according to the Common Terminology Criteria for Adverse Events (CTCAE) version 5.0 (21). Transplant-related mortality (TRM) was defined as death from any cause other than disease progression within 30 days and 100 days post-transplantation (20).

Infectious fever was defined as a single oral temperature of 38.3 °C or higher, or 38.0 °C or higher sustained for 1 hour or more. For all patients with infectious fever, blood cultures were performed. Metagenomic next-generation sequencing (mNGS) was added at the discretion of the treating physician when initial blood cultures were negative but clinical suspicion of infection remained high, or when the patient was not responding to empirical antibiotics. This diagnostic approach was consistent across both groups and reflected standard institutional practice.

Transfusion and G-CSF utilization: Red blood cell and platelet transfusion units, as well as total G-CSF dose administered, were recorded from the medical records. One unit of platelets was defined as a single-donor apheresis unit.

Hospitalization outcomes: Duration of hospitalization was calculated from admission for conditioning to discharge. Total hospitalization costs (in Chinese Yuan, CNY) were extracted from the hospital billing system. For international readership, costs are also reported in US dollars (USD) using the average exchange rate for 2025 (1 USD = 7.2 CNY). Total hospitalization costs included medication costs, transfusion costs, laboratory investigations, nursing and medical staffing, room charges, and medical consumables. Equipment acquisition and maintenance costs were not included, as these are capital expenditures not directly billed to patients.

### 2.6 Statistical analysis

Continuous variables are presented as mean ± standard deviation (SD) or median with interquartile range (IQR), as appropriate, and were compared using the Student’s t-test or Mann-Whitney U test, depending on normality. Normality was assessed using the Shapiro-Wilk test. Categorical variables are expressed as frequencies and percentages, and were compared using the chi-square test or Fisher’s exact test, as appropriate. The cumulative probability of engraftment was estimated using the Kaplan-Meier method, with differences between groups compared using the log-rank test. Since no patient died before engraftment, death was not a competing event.

For platelet transfusion requirements, the data are count data with overdispersion. We therefore used negative binomial regression as the primary analysis. The model adjusted for age, sex, CD34+ cell dose, conditioning regimen, mobilization strategy, and calendar year. Results are reported as incidence rate ratios with 95% confidence intervals. As an alternative modeling approach, we also performed linear regression on log (1+platelet units). This transformation preserves observations with zero transfusions. However, it changes the scale of the outcome and does not yield a coefficient with a direct clinical interpretation. This analysis is therefore presented only as a secondary check on the direction of the association. It is not a formal sensitivity analysis of the same effect estimate.

For binary outcomes with sufficient event numbers, we performed multivariable logistic regression adjusting for the same covariates. Adjusted effect estimates with 95% confidence intervals are reported. For sepsis, only four events occurred in the entire cohort and all were in Group B. This produced complete separation between groups. Conventional multivariable logistic regression would therefore be unstable and potentially overfitted. Accordingly, sepsis was not analyzed with multivariable adjustment and is reported descriptively. More generally, for outcomes with fewer than ten events, multivariable modeling was not performed.

All statistical tests were two-sided, and a *P* value below 0.05 was considered statistically significant. Statistical analyses were performed using SPSS version 26.0 (IBM Corp, Armonk, NY, USA).

## 3 Results

### 3.1 Baseline characteristics

A total of 151 patients with multiple myeloma who underwent autologous stem cell transplantation at our center between February 2022 and March 2026 were enrolled in this study, including 91 patients in Group A (air-flow bed) and 60 patients in Group B (conventional laminar airflow room). The two groups were well balanced with respect to age (55.95 ± 5.67 years vs. 57.45 ± 7.58 years, P = 0.1655), sex (male: 50 [54.9%] vs. 39 [65.0%], P = 0.2404), myeloma subtype (P = 0.5579), ISS stage (P = 0.5037), R-ISS stage (P = 0.9130), and pre-transplantation response (CR: 47 [51.6%] vs. 32 [53.3%]; VGPR: 31 [34.1%] vs. 19 [31.7%]; PR: 13 [14.3%] vs. 9 [15.0%], P = 0.9535).

The distribution of patients by calendar year is shown in **Table 1**. From 2022 to 2024, air-flow beds were the predominant protective environment, with 91 patients managed in air-flow beds and only 7 patients in conventional laminar airflow rooms during this period. Following the completion of new transplant wards in 2025, all subsequent patients (53 patients) were managed in conventional laminar airflow rooms. This phased distribution reflects the availability of transplant facilities rather than patient selection.

Significant intergroup differences were observed in mobilization regimen, conditioning regimen, and infused CD34+ cell dose. Steady-state mobilization was more frequently used in Group B (57 [95.0%] vs. 69 [75.8%], P = 0.0016), whereas chemotherapy-based mobilization was more common in Group A (22 [24.2%] vs. 3 [5.0%]). Regarding conditioning regimens, melphalan alone was administered to 71 patients (78.0%) in Group A and 57 patients (95.0%) in Group B, while melphalan plus bendamustine was used in 20 patients (22.0%) in Group A and 3 patients (5.0%) in Group B (P = 0.0048). Among patients receiving melphalan alone, 55 patients (77.5%) in Group A and 43 patients (75.4%) in Group B received Mel200, while 16 patients (22.5%) in Group A and 14 patients (24.6%) in Group B received Mel140 (P = 0.7940). Accordingly, the CD34+ cell dose was significantly higher in Group A than in Group B (6.15 ± 4.06×10^6^/kg vs. 4.90 ± 2.26×10^6^/kg, P = 0.0318) (**Table 1**).

### 3.2 Engraftment outcomes

All 151 patients achieved successful engraftment. The median time to neutrophil engraftment was 10 days (IQR 10-11) in the overall cohort, with no significant difference between Group A and Group B (10 days [IQR 10-11] vs. 10 days [IQR 10-11], P = 0.6890). The median time to platelet engraftment was also similar between the two groups (12 days [IQR 10-14] vs. 12 days [IQR 10-13], P = 0.1098). The Kaplan-Meier estimated probability of neutrophil engraftment by day 14 was 99.0% in Group A and 100% in Group B. The Kaplan-Meier estimated probability of platelet engraftment by day 21 was 99.0% and 100%, respectively. None of these differences reached statistical significance (**Figure 2**).

**Figure 2:**
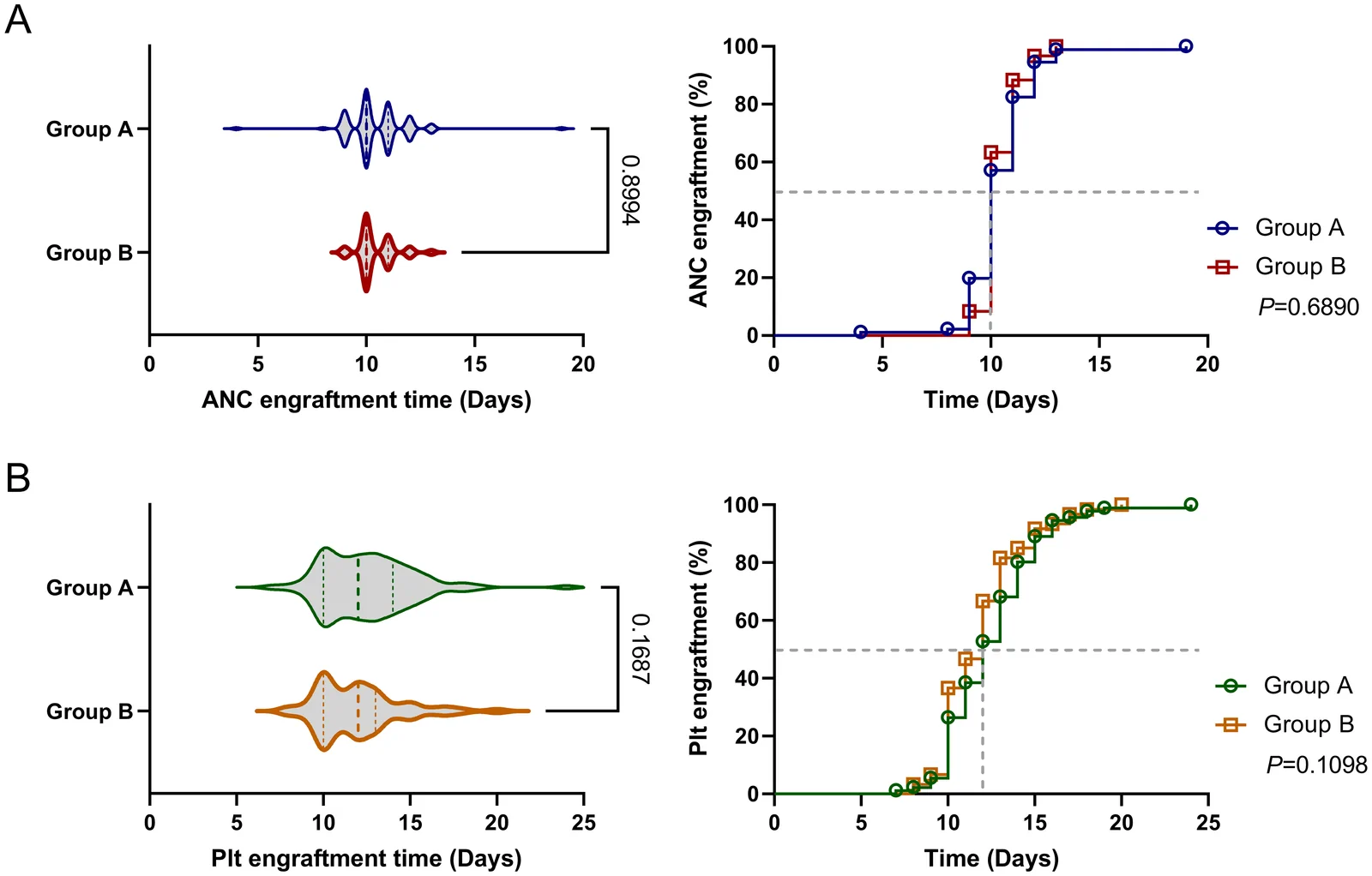
Kaplan-Meier estimated probability of neutrophil and platelet engraftment. **(A)** Neutrophil engraftment, defined as ANC of 0.5×10^9^/L or higher for 3 consecutive days. **(B)** Platelet engraftment, defined as platelet count of 20×10^9^/L or higher without transfusion support for 7 consecutive days. Group A: air-flow bed (n=91); Group B: conventional laminar airflow room (n=60).

### 3.3 Adverse events

One patient in Group B died within 30 days post-transplantation due to severe pulmonary infection and respiratory failure, yielding a 30-day and 100-day TRM rate of 1.7% (1/60), whereas no TRM occurred in Group A.

In the entire cohort, the most common non-hematologic adverse events of any grade were mucositis (134 cases, 88.7%), diarrhea (115 cases, 76.2%), nausea/vomiting (100 cases, 66.2%), hypokalemia (97 cases, 64.2%), and hypoalbuminemia (92 cases, 60.9%). Grade 3 or higher events occurred in 18 cases (11.9%), 18 cases (11.9%), 9 cases (6.0%), 54 cases (35.8%), and 0 cases, respectively. Infection-related events included infectious fever (93 cases, 61.6%, 1 grade 3 or higher), pneumonia (11 cases, 7.3%, all grade 3 or higher), and sepsis (4 cases, 2.6%, all grade 3 or higher). Hemorrhagic events, arrhythmias, and liver and renal function abnormalities were infrequent and generally mild.

Between-group comparisons showed that Group A had lower rates of any-grade mucositis (83.5% vs. 96.7%), diarrhea (67.0% vs. 90.0%), nausea/vomiting (59.3% vs. 76.7%), hypokalemia (57.1% vs. 75.0%), and hypoalbuminemia (56.0% vs. 68.3%). Sepsis occurred exclusively in Group B (4 cases, 6.7%) and was not observed in Group A (0 cases, 0%). Because only four sepsis events occurred and all were in Group B, with complete separation between groups, this comparison is reported descriptively without multivariable adjustment (**Table 2**).

**Table 2:** Adverse events in the whole cohort and by group.

Adverse events	Whole cohort (n=151)		Group A (n=91)		Group B (n=60)	
	All grades	≥ Grade 3	All grades	≥ Grade 3	All grades	≥ Grade 3
Mucositis	134 (88.7%)	18 (11.9%)	76 (83.5%)	8 (8.8%)	58 (96.7%)	10 (16.7%)
Diarrhea	115 (76.2%)	18 (11.9%)	61 (67.0%)	7 (7.7%)	54 (90%)	11 (18.3%)
Infection						
Infectious fever	93 (61.6%)	1 (0.7%)	56 (61.5%)	1 (1.1%)	37 (61.7%)	0
Pneumonia	11 (7.3%)	11 (7.3%)	7 (7.7%)	7 (7.7%)	4 (6.7%)	4 (6.7%)
Sepsis	4 (2.6%)	4 (2.6%)	0	0	4 (6.7%)	4 (6.7%)
Hemorrhage						
Gastrointestinal bleeding	5 (3.3%)	1 (0.7%)	2 (2.2%)	0	3 (5%)	1 (1.7%)
Hematuria	2 (1.3%)	0	2 (2.2%)	0	0	0
Thromboembolism	3 (2.0%)	1 (0.7%)	2 (2.2%)	0	1 (1.7%)	1 (1.7%)
Nausea/Vomiting	100 (66.2%)	9 (6.0%)	54 (59.3%)	4 (4.4%)	46 (76.7%)	5 (8.3%)
Electrolyte abnormality						
Hypokalemia	97 (64.2%)	54 (35.8%)	52 (57.1%)	30 (33.0%)	45 (75.0%)	24 (40.0%)
Hypocalcemia	48 (31.8%)	2 (1.3%)	32 (35.2%)	1 (1.1%)	16 (26.7%)	1 (1.7%)
Arrhythmia						
Sinus tachycardia	9 (6.0%)	0	4 (4.4%)	0	5 (8.3%)	0
Atrial fibrillation	3 (2.0%)	1 (0.7%)	1 (1.1%)	0	2 (3.3%)	1 (1.7%)
Atrial flutter	1 (0.7%)	1 (0.7%)	1 (1.1%)	1 (1.1%)	0	0
Liver function abnormality						
ALT increased	30 (19.9%)	0	16 (17.6%)	0	14 (23.3%)	0
AST increased	52 (34.4%)	2 (1.3%)	27 (29.7%)	0	25 (41.7%)	2 (3.3%)
Blood bilirubin increased	26 (17.2%)	0	10 (11.0%)	0	16 (26.7%)	0
Renal function abnormality						
Creatinine increased	20 (13.2%)	0	10 (11.0%)	0	10 (16.7%)	0
Hypoalbuminemia	92 (60.9%)	0	51 (56.0%)	0	41 (68.3%)	0

Data are presented as n (%). Adverse events were graded according to the Common Terminology Criteria for Adverse Events (CTCAE) version 5.0. Group A: air-flow bed (n=91); Group B: conventional laminar airflow room (n=60). ALT, alanine aminotransferase; AST, aspartate aminotransferase.

Among 93 patients with infectious fever, 14 had pathogens identified by blood cultures and metagenomic next-generation sequencing. In Group A, 56 febrile patients underwent testing (all 56 with blood cultures, and 12 also underwent mNGS); in Group B, 37 febrile patients underwent testing (all 37 with blood cultures, and 8 also underwent mNGS). The overall pathogen detection rate was 15.1%. The detection rate was lower in Group A than in Group B (5.5% [5/91] vs. 15.0% [9/60], P = 0.050). The pathogen spectrum also differed between groups. Group A was predominantly Gram-negative bacilli, with Citrobacter freundii as the most common isolate, whereas Group B exhibited a broader spectrum including Gram-positive cocci, Gram-negative bacilli, anaerobes, and viruses, with polymicrobial infections in 5 of 9 cases (55.6%). High-risk multidrug-resistant organisms, including Acinetobacter baumannii, Enterococcus faecium, and Staphylococcus aureus, were isolated exclusively in Group B.

### 3.4 Transfusion support and G-CSF administration

Red blood cell transfusions were administered at a median of 0 units (IQR 0-0) in both Group A and Group B, with no significant difference between the two groups (P = 0.8342). For platelet transfusions, Group A required a median of 2 units (IQR 1-3) compared with 4 units (IQR 3-5) in Group B (P<0.0001). The total G-CSF dose administered was comparable between the two groups, with a median of 3150 μg (IQR 2850-3750) in Group A and 3375 μg (IQR 3150-3600) in Group B (P = 0.9626) (**Figure 3**).

**Figure 3:**
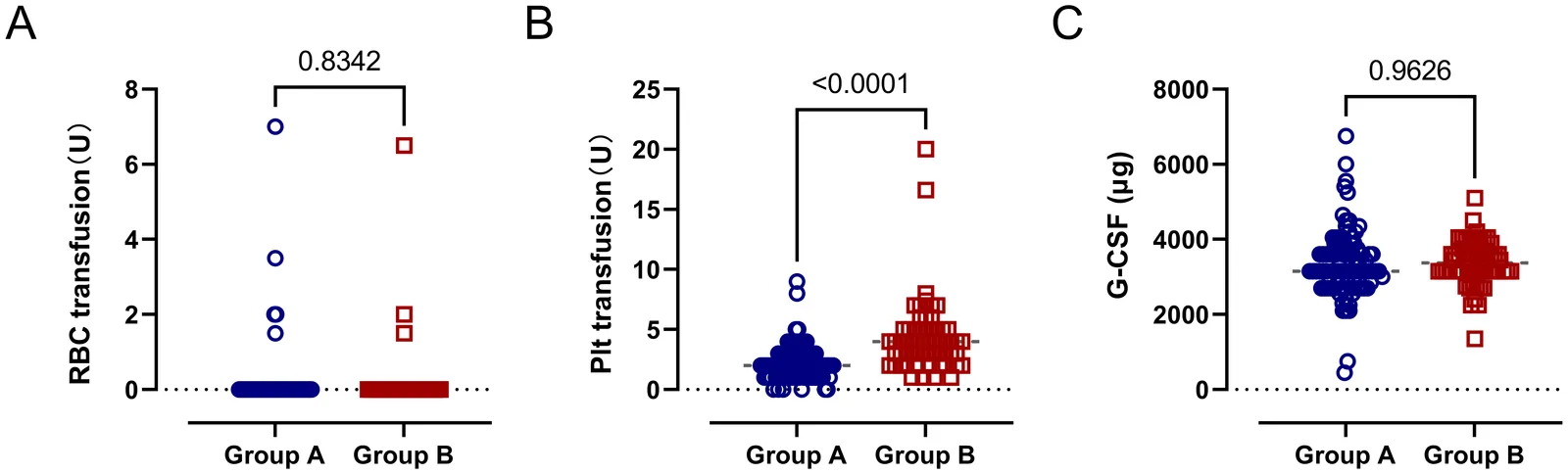
Transfusion requirements and G-CSF utilization. **(A)** Red blood cell transfusion units. **(B)** Platelet transfusion units. **(C)** Total G-CSF dose administered. Boxes represent the interquartile range (IQR), horizontal lines indicate the median, whiskers denote the range, and dots represent individual outliers. Group A: air-flow bed (n=91); Group B: conventional laminar airflow room (n=60).

### 3.5 Hospitalization duration and costs

The median duration of hospitalization was 30 days (IQR 26-35) in Group A and 28 days (IQR 24-35) in Group B, with no significant difference between the two groups (P = 0.1285). Regarding total hospitalization costs, the median expense was 121,738 CNY (IQR 97,316-146,006; approximately $16,908, IQR $13,516-$20,279) in Group A compared with 137,506 CNY (IQR 122,982-155,373; approximately $19,098, IQR $17,081-$21,580) in Group B (P = 0.0002) (**Figure 4**).

**Figure 4:**
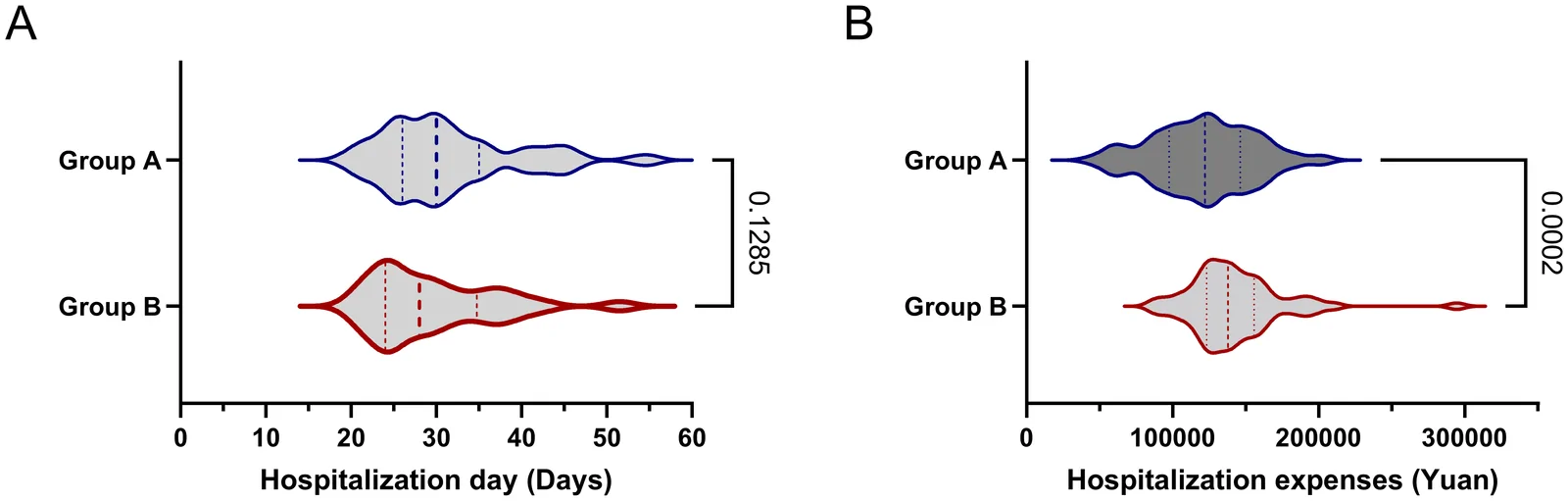
Hospitalization Duration and Costs. **(A)** Duration of hospitalization (days). **(B)** Total hospitalization expenses (Chinese Yuan, CNY). Group A: air-flow bed (n=91); Group B: conventional laminar airflow room (n=60).

### 3.6 Multivariable analysis

To address potential confounding from imbalances in baseline characteristics and temporal trends, we performed multivariable regression analyses. For platelet transfusion requirements, the data are count data with overdispersion. We therefore used negative binomial regression as the primary analysis. Air-flow bed use was independently associated with a lower number of platelet transfusion units after adjusting for age, sex, CD34+ cell dose, conditioning regimen, mobilization strategy, and calendar year (IRR = 0.50, 95% CI: 0.40 to 0.63, P<0.001). Higher CD34+ cell dose was also independently associated with fewer platelet transfusions (IRR = 0.94 per 1×10^6^/kg increase, 95% CI: 0.90 to 0.98, P = 0.003). The air-flow bed effect persisted after accounting for this and other covariates.

In an alternative linear regression model using log (1+platelet units), the direction of the association was consistent (adjusted β=-1.46, 95% CI: -2.15 to -0.77, P<0.001). Because the log (1+units) transformation changes the scale of the outcome, this coefficient does not have a direct clinical interpretation. This result should therefore be regarded as a secondary directional check and not as a formal sensitivity analysis of the same effect estimate.

For binary outcomes, multivariable logistic regression was performed only for infectious fever and pathogen detection. Event numbers were sufficient for these outcomes. For infectious fever and pathogen detection, the point estimates suggested lower risk in Group A. The confidence intervals crossed unity after adjustment. Infectious fever showed an adjusted OR of 0.68 (95% CI: 0.32 to 1.45, P = 0.32). Pathogen detection showed an adjusted OR of 0.42 (95% CI: 0.12 to 1.48, P = 0.18). For sepsis, only four events occurred and all were in Group B. This produced complete separation between groups. Multivariable logistic regression was therefore not performed. The comparison is reported descriptively as 0/91 (0%) in Group A versus 4/60 (6.7%) in Group B. No adjusted effect estimate is presented for sepsis. These adjusted results should be interpreted cautiously given the limited number of events for some outcomes.

## 4 Discussion

In this retrospective cohort study, we found that ASCT performed in air-flow beds achieved engraftment outcomes comparable to those in conventional laminar airflow rooms, with a favorable safety profile characterized by lower rates of mucositis, diarrhea, and sepsis, a narrower pathogen spectrum, reduced platelet transfusion requirements (independently associated with air-flow bed use in multivariable analysis), and substantial cost savings.

The baseline characteristics between the two groups were not entirely balanced. Patients in Group A more frequently received chemotherapy-based mobilization and a melphalan plus bendamustine conditioning regimen, which resulted in a significantly higher CD34+ cell dose. These factors, particularly the intensified conditioning, would conventionally be expected to increase mucosal damage and gastrointestinal toxicity. Yet despite this unfavorable baseline profile, Group A paradoxically exhibited lower rates of mucositis, diarrhea, and sepsis. This counterintuitive finding may suggest that the protective environment of the air-flow bed could offset some of the added toxicity burden from more intensive conditioning. However, given the observational nature of this study and the multiple confounding factors, we cannot establish causality, and alternative explanations including differences in supportive care or unmeasured confounders cannot be excluded.

The comparable engraftment outcomes between groups are consistent with prior studies of alternative protective environments and underscore that engraftment is primarily determined by CD34+ cell dose and graft quality rather than the specific type of protective environment (15, 16). The absence of engraftment failure in either group further supports that air-flow beds provide sufficient environmental protection during the neutropenic period.

The infection profile in our cohort reveals several patterns. Although the overall incidence of infectious fever was comparable between groups, the pathogen detection rate was lower in Group A (5.5% vs. 15.0%, P = 0.050), and the pathogen spectrum in Group A was notably narrower, predominantly comprising gut-derived Gram-negative bacilli. This pattern suggests that infections in air-flow bed patients may be primarily driven by bacterial translocation across damaged intestinal mucosa following conditioning chemotherapy, rather than by environmental acquisition. In contrast, the broader and more complex pathogen spectrum observed in Group B, including high-risk multidrug-resistant organisms such as Acinetobacter baumannii, Enterococcus faecium, and Staphylococcus aureus, could raise the possibility of healthcare-associated environmental contamination. However, we acknowledge that this interpretation remains speculative without environmental microbiological surveillance or molecular epidemiological evidence. The small number of microbiologically documented infections also limits this interpretation.

The occurrence of sepsis exclusively in Group B is notable but must be interpreted with caution. Only four sepsis events occurred in the entire cohort and all were in Group B. This produced complete separation between groups. The small number of events precludes reliable multivariable adjustment. The descriptive comparison cannot establish an independent protective effect of air-flow beds. We therefore report this finding as a hypothesis-generating observation rather than as a confirmed association.

The significantly lower platelet transfusion requirement in Group A (median 2 vs. 4 units, P < 0.0001) represents a clinically meaningful benefit. In negative binomial regression, air-flow bed use was independently associated with a 50% lower number of platelet transfusion units after adjusting for CD34+ cell dose and other covariates. Higher CD34+ cell dose was also independently associated with fewer platelet transfusions. Because platelet transfusion units are count data with overdispersion, negative binomial regression provides a more appropriate and interpretable estimate than linear regression on log-transformed values. This reduction decreases transfusion-related risks and costs. It also alleviates the burden on blood bank resources. The comparable red blood cell transfusion needs and G-CSF utilization between groups further support that air-flow beds do not compromise the overall quality of transplant care.

The economic advantage of air-flow beds is substantial. The median total hospitalization cost in Group A was ¥121,738 (approximately $16,908), representing an approximately 11.5% reduction compared with Group B (¥137,506, approximately $19,098, P = 0.0002). Because equipment acquisition and maintenance costs were not included in the hospitalization-cost endpoint, the observed difference cannot be attributed to lower capital or maintenance costs of air-flow beds. The difference is more likely explained by the components actually included in total hospitalization costs, particularly the reduced platelet transfusion requirements in Group A. Lower infrastructure costs of air-flow beds may represent an additional economic advantage. However, this is distinct from the hospitalization-cost difference demonstrated in the present analysis and was not directly measured here. Given that hospitalization duration was comparable between groups, air-flow beds offer a practical and scalable solution to expand transplant access in resource-limited settings. This applies where conventional laminar airflow rooms are unavailable or insufficient. Moreover, the ability to accommodate family members throughout hospitalization in Group A may provide meaningful psychosocial support, an important but often overlooked aspect of transplant care that conventional isolation rooms inherently preclude.

Several limitations should be acknowledged. First, and most importantly, protective environment and transplant era were strongly intertwined. From 2022 to 2024, 91 of 98 transplantations used air-flow beds. All 53 transplantations in 2025 and 2026 used conventional laminar airflow rooms. Although we included calendar year as a covariate in multivariable analyses, statistical adjustment cannot fully resolve this near-collinearity or exclude unmeasured changes over time in clinical practice, supportive care, or infection epidemiology. The observed associations, particularly those involving supportive-care and infectious outcomes, therefore cannot be confidently separated from a potential era effect. Second, baseline differences in mobilization regimens, conditioning regimens, and CD34+ cell doses represent important confounders. We adjusted for these in multivariable models, but residual confounding cannot be excluded. Third, the small number of microbiologically documented infections limits the statistical power for comparing pathogen spectra. The sepsis comparison was based on only four events and precluded reliable multivariable adjustment. Fourth, the single-center design may limit the generalizability of our findings to other settings with different infection epidemiology and healthcare systems. Finally, the follow-up duration was insufficient to evaluate long-term outcomes such as progression-free and overall survival.

Future research should prioritize prospective randomized controlled trials comparing air-flow beds with conventional laminar airflow rooms. Patient-reported outcomes and formal psychosocial assessments should be systematically incorporated. Long-term follow-up studies are needed to confirm that the favorable short-term outcomes are maintained over time.

In conclusion, our findings suggest that air-flow beds may represent a safe, effective, and economically viable alternative to conventional laminar airflow rooms for autologous transplantation in multiple myeloma. This approach has the potential to expand transplant access without compromising short-term patient outcomes. However, given the strong intertwining of protective environment with transplant era and the limited number of events for some outcomes, these findings should be regarded as hypothesis-generating. Prospective validation in well-controlled randomized trials is warranted to confirm these observations.

## Data Availability

The raw data supporting the conclusions of this article will be made available by the authors, without undue reservation.
