# Supplementary material for: Efficacy and safety of air-flow beds for autologous hematopoietic stem cell transplantation in multiple myeloma: a retrospective cohort study
Source: Front Oncol. 2026 Sep 30;16:1953570. doi: 10.3389/fonc.2026.1953570 (PMC13661374; doi:10.3389/fonc.2026.1953570)
Supplement: Supplementary file 1 [file Supplementaryfile1.docx]

**Efficacy and safety of air‑flow beds for autologous hematopoietic stem cell transplantation in multiple myeloma: a retrospective cohort study**

**Supplementary Figure 1.** Photograph of the air-flow bed system in a standard hospital ward. The air-flow bed is placed in a standard single-occupancy hospital room. Key visible components include the HEPA filtration unit mounted at the top of the canopy, the transparent isolation canopy surrounding the patient area, and the bed frame. The system does not require dedicated laminar flow ward infrastructure. Family members may remain outside the canopy during hospitalization.
